# Unlocking the novel activation mechanism of human IL-18

**DOI:** 10.7555/JBR.38.20240154

**Published:** 2024-06-17

**Authors:** Yingchao Hu, Yuxian Song, Shuo Yang

**Affiliations:** 1 The Affiliated Wuxi People's Hospital of Nanjing Medical University, Wuxi People's Hospital, Wuxi Medical Center, Department of Immunology, State Key Laboratory of Reproductive Medicine and Offspring Health, Jiangsu Key Lab of Cancer Biomarkers, Prevention and Treatment, Collaborative Innovation Center for Personalized Cancer Medicine, Nanjing Medical University, Nanjing, Jiangsu 211166, China; 2 Department of Immunology, School of Basic Medical Sciences, Nanjing Medical University, Nanjing, Jiangsu 211166, China

Interleukin (IL)-18, a member of the IL-1 family, is commonly known as an interferon-γ inducer and is expressed in both hematopoietic and non-hematopoietic cells, such as intestinal epithelial cells, keratinocytes, and endothelial cells. In the immune system, the mature IL-18 plays a critical role in eliminating tumors and infectious agents by activating NK cells and T-lymphocytes, and by synergizing with other cytokines like IL-12 and IL-1β to induce inflammation^[1–2]^. However, excessive activation of IL-18 may cause a variety of immune-inflammatory diseases, including psoriasis, atherosclerosis, Alzheimer's disease, and inflammatory bowel disease^[3–4]^. Regarding the activation mechanism of IL-18, similar to immune cells, mouse non-immune cells rely on caspase-1 for its processing and maturation. However, the activation mechanism of IL-18 in human non-immune cells remains unclear.

It is generally recognized that pro-IL-1β and pro-IL-18, two crucial proinflammatory factors, are cleaved and activated by caspase-1 in the canonical inflammasome pathway^[5]^. Nevertheless, it remains unknown whether and how these cytokine substrates are recognized and activated by caspase-4/5/11 in the non-canonical inflammasome pathway. Unlike the canonical inflammasome pathway, which is primarily confined to innate immune cells like monocytes and macrophages, the non-canonical inflammasome pathway is prevalent in numerous non-immune cells. Notably, while the processing and maturation of mouse IL-18 in non-immune cells depend on caspase-1, a significant amount of active IL-18 is still widely distributed in some human non-immune cells with low caspase-1 expression, indicating that the cleavage and processing of pro-IL-18 may not entirely rely on caspase-1. In addition, it has been reported that the maturation of pro-IL-18 is associated with the activation of caspase-4 in human gastrointestinal epithelial cells infected with *Salmonella*^[6]^, but whether caspase-4 can directly cleave and activate pro-IL-18 remains unclear. Recently, Shi *et al*^[7]^ published a paper online in the journal *Nature*, systematically elucidating the maturation and activation mechanism of IL-18 during non-canonical inflammasome activation in human cells. The investigators found that pro-IL-18 and caspase-4/5 were widely co-expressed in various human non-immune cell lines, suggesting that the broadly expressed caspase-4/5 may have the ability to process pro-IL-18. Through traditional biochemical strategies and crystal structure analysis, they uncovered that the inflammatory cytokine IL-18 served as a physiological substrate for caspase-4/5 in the non-canonical inflammasome pathway in humans.

To gain a deeper understanding of the recognition mechanism of pro-IL-18 by caspase-4/5, the crystal structure of the complex between caspase-4 and pro-IL-18 was resolved. Structural analysis revealed that caspase-4 recognized pro-IL-18 through two key binding interfaces. The first interface was formed by the tight interaction between the catalytic pocket of caspase-4 and the tetrapeptide LESD of pro-IL-18. The second binding interface was formed by the exosite in caspase-4, with its L2/L2′ sheet flanking the outer face of the β1/β4 sheet in pro-IL-18. Upon dual-binding interface recognition, caspase-4 was found to cleave pro-IL-18 and induce conformational changes in cleaved IL-18, including β4 rotation and β5-β9 rearrangement, thus exposing two key sites, α1 and β5-β6, for receptor binding and ultimately transforming IL-18 into its active form.

**Figure 1:**
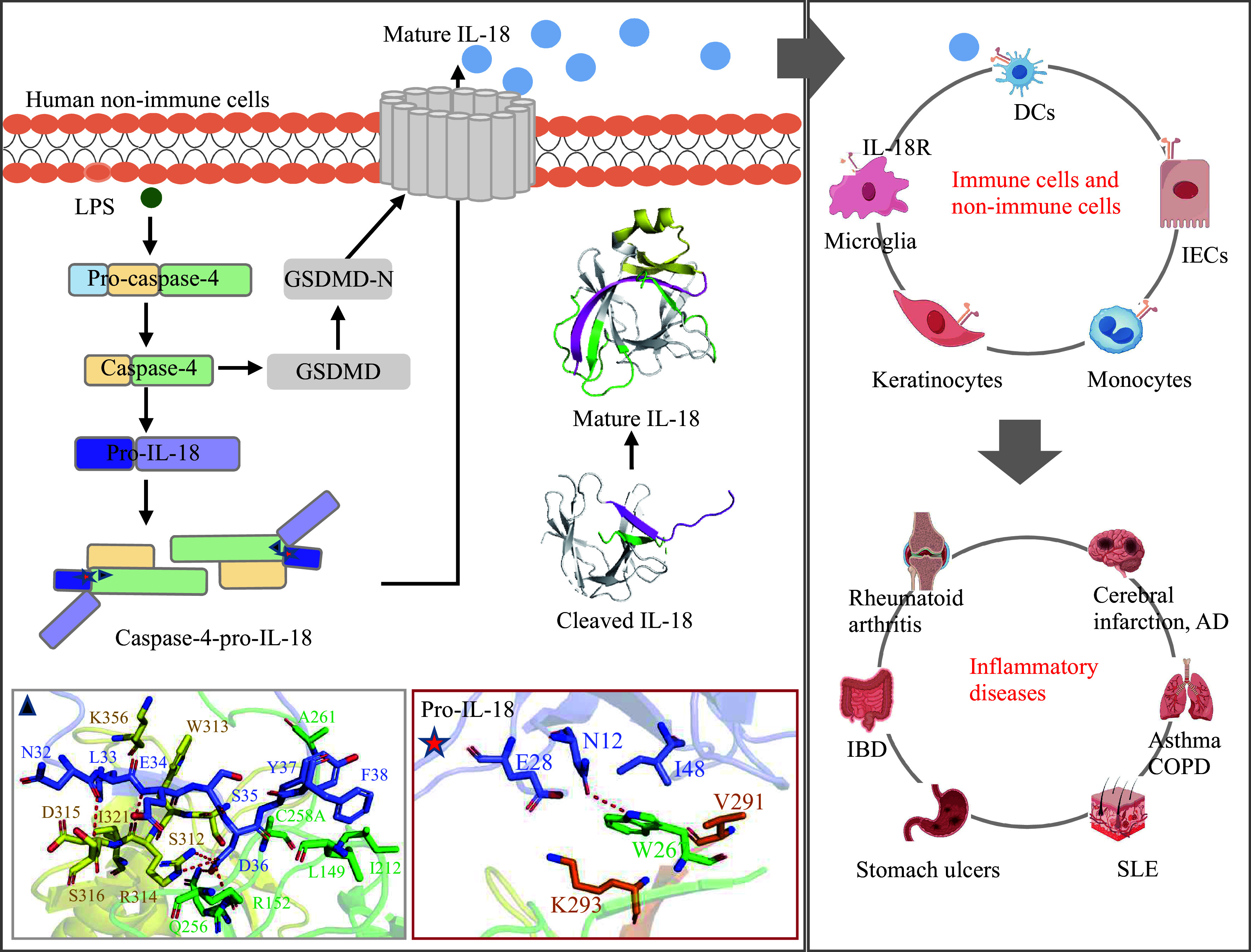
Caspase-4/5 facilitates IL-18 maturation, triggering specific biological responses.

In summary, this study has established that IL-18 serves as a physiological substrate for caspase-4/5 in the non-canonical inflammasome pathway in human cells, and has fully revealed the mechanism by which caspase-4/5 recognize and cleave pro-IL-18 in the innate immune pathway, as well as the molecular pathway underlying the processing and maturation of human pro-IL-18 into a physiologically active cytokine (***Fig. 1***).

This study offers a new perspective on the recognition mechanism of human caspase enzymes for the processing of pro-IL-18 in the innate immune pathway, and provides sufficient evidence and novel ideas for the development of drugs targeting the caspase-4-IL-18 pathway. Mature IL-18 regulates innate and adaptive immune responses by binding to its receptor and mediating inflammatory responses. However, excessive activation of IL-18 may lead to autoimmune or inflammatory diseases. Therefore, effectively suppressing the overactivation of this pathway is an urgent issue that needs to be addressed. Notably, the activated caspase-3 has been reported to cleave and inactivate IL-18^[8–9]^, indicating that the caspase-4-IL-18 axis may be negatively regulated by caspase-3. In addition, this study revealed that murine caspase-11 acquired the ability to cleave pro-IL-18 after replacing its L4 with the corresponding amino acid from caspase-4, which suggests that L4 of caspase-4 may serve as a potential drug target for developing effective treatment strategies against IL-18-related autoimmune diseases. It is worth mentioning that at the same time, Devant *et al*^[10]^ published an online paper entitled "Structural Insights into Cytokine Cleavage by Inflammatory Caspase-4" in the journal *Nature*, also revealing the mechanism by which caspase-4 cleaves and activates pro-IL-18.
